# Advancing equity in applied health and care research training: a reflexive perspective on the NIHR INSIGHT programme

**DOI:** 10.3389/fmed.2026.1803057

**Published:** 2026-06-29

**Authors:** Nagina Khan, Nina Stewart, Kish Bhatti-Sinclair, Stephen Peckham

**Affiliations:** 1 Centre for Health Services Studies, University of Kent, Canterbury, United Kingdom; 2 School of Education, Sport and Health Sciences, University of Brighton, Brighton, United Kingdom; 3 Department of Childhood, Social Work and Social Care, University of Chichester, Chichester, United Kingdom

**Keywords:** applied health research training, critical pedagogy, decolonising pedagogy, equity-oriented education, inclusive curriculum, patient and public involvement and engagement (PPIE), social accountability, structural competence

## Abstract

Health inequities in the United Kingdom disproportionately affect racialised, globally marginalized, and working-class communities. Research training programmes shape who becomes a researcher, which questions are prioritized and which knowledge is recognized as legitimate. This Perspective provides a reflexive, practice-informed account of the NIHR Inspiring Students into Research Training (INSIGHT) Programme across three South East England institutions: the University of Kent (MSc Applied Health and Care Research), the University of Brighton (MRes), and the University of Chichester (MRes). Drawing on programme documentation, early evaluation insights, teaching experiences and reflections from programme leaders, learners, and partner organizations, the paper examines how equity-oriented pedagogies have been embedded in postgraduate research training. Illustrative reflections from learners and informal contributors, drawn from module feedback, demonstrate the programme’s collaborative ethos. The curriculum integrates structural competence, cultural humility, epistemic justice and co-produced patient and public involvement (PPI) alongside core research methods. Findings suggest that embedding equity-focused, socially accountable approaches enhances learner engagement, supports epistemic inclusion, and strengthens the relevance of research for underserved communities. The programme also highlights structural tensions between workforce composition and eligibility criteria: many internationally trained and minoritised practitioners in the NHS and social care, often recruited to address workforce shortages, may be excluded from funded training due to residency, fee-status, or professional timelines. This Perspective argues that reflexive, practice-informed curriculum design, together with more flexible and inclusive eligibility policies, can contribute to a diverse, socially accountable applied health and care research workforce.

## Introduction

This article presents a reflexive, practice-informed Perspective based on the authors’ roles as educators and programme leads within the National Institute of Health Research (NIHR) Inspiring Students into Research Training (INSIGHT) Programme (1). Rather than reporting a formal programme evaluation, it reflects on curriculum design, pedagogical choices and observed shifts in learning environments, drawing on programme documentation, informal learner feedback and professional experience. The focus is on equity and access within postgraduate applied health and care research training, with particular attention to how curriculum design and funding structures influence who can engage with research pathways. While decolonising scholarship informs the pedagogical approach, this paper does not aim to evaluate the “decolonisation of the workforce”; instead, it examines how equity-oriented educational practices can widen participation, surface structural barriers and support socially accountable research training within existing systems.

Decolonising health professions education (HPE) is increasingly recognized as essential to addressing structural inequities in healthcare and developing a research workforce capable of responding to the needs of diverse communities (2, 3). In this context, decolonising approaches are considered one component of a broader equity-focused agenda that seeks to dismantle systemic barriers in research training. Traditional programmes often remain grounded in Western epistemologies, deficit-oriented views of communities and narrow notions of legitimate evidence, which can inadvertently reproduce inequities in whose knowledge is valued and whose health outcomes are prioritized (4–6). Embedding decolonising principles in HPE expands recognition of diverse knowledge systems, fosters cultural humility and structural competence and equips researchers to engage meaningfully with historically marginalized populations.

Major funders, including the NIHR, increasingly prioritize programmes that enhance inclusivity, representation, and research impact across diverse communities (7). The INSIGHT Programme was established to support early-career practitioners from underrepresented professions by providing fully funded MSc and MRes places, including tuition fees and stipends. Beyond developing research skills, the programme nurtures interest in research careers through mentorship, structured pathways and training that promotes participation and success in applied health and care research.

The INSIGHT programmes at the University of Kent, University of Brighton, and University of Chichester explicitly embed social justice, structural competence, and equity principles into postgraduate health research education. By targeting practitioners from diverse NHS and social care settings including racialised, working-class, or globally marginalized communities the programmes leverage lived experience to inform research on health inequities, community-based care and culturally responsive practice. Through the combination of NIHR financial support and curricula emphasizing equity, participatory methods such as patient and public involvement (PPI), and reflexive practice, the MSc/MRes programmes seek to transform individual careers while influencing institutional and systemic approaches to research training.

This Perspective draws on programme documentation, curriculum materials, learner feedback, informal feedback, and the authors’ positionality as programme leads and educators. It highlights opportunities, tensions and constraints encountered in widening participation, shaping inclusive learning environments and supporting socially accountable research within existing structural frameworks. The authors are educators and programme leads within the NIHR INSIGHT initiatives described in this paper. Our reflections therefore draw on insider perspectives shaped by programme leadership, curriculum design, and teaching practice, which we approach reflexively to critically examine both opportunities and constraints within the programme.

## Programme overview

The NIHR INSIGHT Programme is a nationally funded initiative designed to develop applied health and care research capacity among early-career practitioners by integrating postgraduate research training with practice-based experience (1). Operating across multiple regions in England, the programme seeks to widen participation in research careers by supporting practitioners from professions and backgrounds historically underrepresented in health and care research, including racialised, working-class and globally marginalized communities (8, 9). Participating institutions include the University of Kent, the University of Brighton, and the University of Chichester, each offering fully funded Master’s-level programmes (MSc/MRes) that cover tuition fees and provide a stipend, enabling learners to engage in research alongside professional practice (10).

At the University of Kent, the MSc in Applied Health and Care Research is delivered as a practice-based, equity-focused programme. It recruits practitioners from a broad range of NHS, social care, public health, and third-sector settings across the South East of England, with an explicit emphasis on diversity and inclusion. Eligibility criteria require participants to be within 5 years of completing their initial professional training, registered with a recognized health or social care regulatory body, and employed within an NHS, local authority, or voluntary sector organization. By combining targeted eligibility criteria with financial support, the programme cultivates a research-capable workforce that reflects the diversity of contemporary health and care systems (11).

The University of Brighton offers the INSIGHT MRes in Health and Care Research as a research-intensive route, supporting early-career practitioners to develop applied research capability alongside professional practice. The programme emphasizes the design, conduct and dissemination of independent research projects relevant to health, public health and social care contexts. Supervised research, methodological training and engagement with local NHS and community partners help participants build confidence in applied research and explore progression to doctoral study and research leadership roles (8).

At the University of Chichester, the INSIGHT MRes combines foundational research methods teaching with a substantial independent research project grounded in learners’ professional contexts. The programme emphasizes applied, service-relevant research that responds to local and regional health and care priorities, supporting practitioners to integrate research into clinical, organizational and community settings. Through this approach, learners develop skills to translate research findings into practice, inform service improvement, and influence policy (9).

Across all three institutions, the curriculum integrates co-produced learning, reflective practice and participatory approaches such as patient and public involvement (PPI), alongside core methodological training in applied health and care research (1). Participants are supported to develop structural competence, cultural humility, and equity-oriented research skills, while engaging with faculty, peers and collaborative projects. Learners are encouraged to critically interrogate the politics of knowledge production, examine structural inequities in health and care systems, and adopt culturally responsive approaches to applied research (11).

Collectively, the INSIGHT programmes at Kent, Brighton, and Chichester aim to broaden access to research career pathways, strengthen applied research capacity, and enhance the relevance and impact of research in diverse communities. By linking academic training with NHS, social care, and voluntary sector organizations, the programmes provide a pathway for practitioners to address health and social inequities and embed socially accountable research practices within professional practice (10).

## Equity and access challenges

A central challenge within the NIHR INSIGHT Programme relates to equity and access. While the programme’s eligibility criteria are designed to align funded training with workforce development priorities, these same criteria can generate unintended exclusions (8, 9). To be eligible, applicants must:

- Be within 5 years of completing professional training in a regulated applied health or social care profession.
- Be employed by an NHS, local authority, or third-sector organization.
- Hold professional registration with a recognized regulatory body, such as the Nursing and Midwifery Council (NMC) or Health and Care Professions Council (HCPC) (10).

These criteria are intended to support early-career practitioners embedded within the UK health and care system and to ensure that public investment is directed toward those positioned to contribute directly to service improvement. However, they intersect unevenly with contemporary workforce realities (11). In particular, the 5-year post-qualification requirement and associated fee-status and residency regulations can disproportionately affect internationally trained and minoritised practitioners, who may be actively recruited into NHS and social care roles yet fail to meet programme eligibility thresholds despite substantial professional experience (8, 11).

This creates a structural tension between workforce policy, which increasingly relies on globally mobile practitioners to address staffing shortages, and research training funding mechanisms, which remain narrowly defined around conventional professional timelines. Consequently, practitioners whose lived experience and positionality could meaningfully inform equity-oriented, community-relevant, and applied research may be excluded from funded research training pathways (11).

Addressing these misalignments is essential if initiatives such as the NIHR INSIGHT Programme are to advance equity, representation, and social accountability in applied health and care research (1, 8). Embedding flexible, inclusive approaches to eligibility and funding could enable a more diverse and socially accountable research workforce capable of responding to complex health inequities and community needs.

## Curriculum innovations and pedagogical approaches

The NIHR INSIGHT MSc and MRes programmes are deliberately designed to foreground equity-oriented approaches within applied health and care research training. The curriculum integrates traditional research methods with practice-based learning, reflexive exercises, and participatory pedagogies, encouraging students to critically examine the social, historical, and structural contexts that shape health inequities (8, 9).

Drawing on module evaluations, reflective assessments, and programme team reflections, students are introduced to core methodological competencies alongside structured opportunities to interrogate power, positionality, and whose knowledge counts in research. For example, reflective assessments and learning journals invite students to situate their research interests within their own professional and community contexts. This practice encourages critical engagement with lived experience as a source of insight rather than bias, promoting research approaches that are socially accountable and responsive to community needs.

Feedback from students highlighted the impact of these pedagogical innovations. Participants reported that reflective exercises, PPI-focused activities, and case-study discussions enhanced their understanding of decolonising research principles and helped them articulate practitioner-informed research questions confidently. One student noted that activities focused on researcher positionality “moved qualitative research from being a neutral data collection exercise to a deeply social process where my own background and power as a researcher directly impact the study.” Another highlighted the value of guest lectures and participatory sessions, which reinforced awareness of structural inequities and culturally responsive research practice.

Collectively, these innovations demonstrate that small, intentional pedagogical changes such as integrating reflective practice, participatory approaches and critical discussion can meaningfully shape access, participation and the classroom climate. They provide early-career practitioners with tools to conduct applied research that is ethically informed, socially accountable and inclusive of diverse epistemologies, aligning with the broader goals of decolonising health research education (1, 11).

## Co-produced learning and PPI

Co-produced learning is a core feature of the INSIGHT curriculum, operationalising principles of patient and public involvement (PPI) within applied health and care research training. Students engage with the PPI lead with lived experience, to undertake a workshop in co-design learning activities, and project consultations, enabling them to apply structural competence and cultural humility to real-world research questions (1, 12).

Formative feedback, reflective assignments and programme team reflections indicate that participatory approaches challenge traditional hierarchies of expertise and strengthen the ethical and contextual relevance of research. Student reflections highlighted the value of PPI-focused learning: one participant noted that the PPI session with the PPI lead had directly influenced their research design, providing confidence to integrate patient perspectives into their own projects. Another described co-production activities as transformative for understanding researcher positionality, stating that these exercises encouraged them to critically consider how power and lived experience shape research processes.

From an educational perspective, co-produced learning functions as a site of reflexive pedagogy, prompting educators to question assumptions about knowledge ownership, the role of expertise and the ways in which lived experience informs research (13). These reflections have guided iterative adaptations to curriculum content, assessment design, and teaching methods across successive cohorts, enhancing both student learning outcomes and the practical impact of research projects.

Embedding PPI and co-production within the curriculum aligns with equity-oriented and socially accountable research training, preparing early-career practitioners to design and conduct applied health research that is responsive to community needs, ethically robust and methodologically inclusive (9, 11).

## Decolonising and inclusive content

The INSIGHT curriculum deliberately challenges Western-centric epistemologies by integrating Global South and non-Western perspectives, critical analyses of historical and contemporary inequities, and structured reflective exercises on the politics of knowledge production (9, 14). Reading lists were curated to foreground scholarship authored by diverse researchers and communities, while assessment tasks require students to critically interrogate power, positionality and epistemic authority within their research practice.

At the University of Kent, for instance, modules were revised to include diverse reading materials and guest lectures from researchers working with global health contexts, underserved local communities, and patient or user experts with lived experience. Students are required to formally reflect on their positionality and practice reflexivity within assessed assignments, embedding these skills into learning outcomes and ensuring they are actively evaluated as part of student progression.

Student feedback indicated that these curriculum elements enhance awareness of structural inequities, broaden understanding of knowledge production, and reinforce the practical relevance of research to diverse communities. One student highlighted that the integration of non-Western epistemologies and reflexive exercises helped them “critically question whose knowledge is valued in research,” while another noted that the guest lectures provided tangible examples of equity-focused research in applied health and care settings.

Embedding decolonising and inclusive content in postgraduate research training not only promotes cultural humility and ethical awareness but also equips early-career practitioners to engage in socially accountable research that is responsive to historically marginalized populations (15–17).

## Assessment and feedback innovations

Assessment strategies within the INSIGHT programmes are intentionally designed to mirror professional practice while embedding principles of equity, social justice, and cultural humility. Students receive concise marginal feedback, narrative summaries, and actionable next steps, supporting development of both academic competencies and applied professional skills. Assessment outputs include policy briefs, practice guidelines, and public-facing summaries, encouraging learners to consider diverse audiences and the social relevance of their research.

At the University of Kent, assessment frameworks integrate structured formative feedback, peer-review workshops, and one-to-one supervision. These approaches prompt students to reflect on their positionality, research choices, and engagement with marginalized communities. Feedback explicitly encourages consideration of equity, inclusivity, and culturally responsive research practice, reinforcing that research outcomes must be socially accountable as well as methodologically robust.

Student evaluations indicate that these strategies enhance confidence, promote critical reflexivity, and improve understanding of equitable research practices. One student reported that reflective feedback on a practice-focused assignment helped them “better articulate practitioner-informed research questions while considering the voices and priorities of diverse communities.” Faculty observations suggest that these approaches are transferable across institutional contexts, promoting inclusive assessment design and embedding social justice principles in postgraduate research training more broadly (18, 19).

## Impact on students and research practice

The NIHR INSIGHT Programme demonstrates impact at multiple levels on students, faculty, and the wider healthcare and academic context. Learners develop applied research skills while also building cultural humility, structural competence, and awareness of equity in research and professional practice.

Student feedback highlights meaningful qualitative changes: students from minoritised backgrounds report speaking up more, leading discussions, and contributing perspectives shaped by their lived experience. Engagement with material on how professional norms and disciplines have been historically constructed through colonial and racialised histories encourages critical reflection on systemic inequities. Guest speakers working in global health, underserved communities, and patient-led research inspire students to connect theory with practice and consider the broader social impact of research.

Bringing structural inequities from the literature and the author’s research particularly regarding career progression, research funding and publication opportunities for women of color into classroom discussions allows learners to see and interrogate systemic barriers, linking their professional experience to equity-focused research practice.

These reflective, practice-informed dialogues foster both critical awareness and a sense of responsibility, encouraging students to produce research that is socially accountable, contextually relevant, and responsive to diverse community needs. Collectively, these small but significant shifts in classroom climate, participation, confidence, and reflexive engagement illustrate how an equity-oriented curriculum can influence learning outcomes, professional practice, and the development of research-capable practitioners (20–22).

## Impact, structural insights, lessons learned, and policy implications

The NIHR INSIGHT Programme produces impact at multiple levels, on students, faculty, and institutional practice, by combining equity-focused research training with professional engagement (1). Students from diverse NHS and social care backgrounds, including racialised, working-class, and globally marginalized communities, engage in research projects directly relevant to their professional practice. Co-produced projects with patients, service users, and community stakeholders foster agency, belonging, and confidence to critically examine inequities in healthcare (12). Formative feedback, reflective exercises, and structured supervision support the development of evidence-informed practice, critical analysis, and reflexive engagement (23).

Small but meaningful qualitative shifts indicate programme impact: students from minoritised backgrounds reporting that they speak up more, lead discussions, and bring perspectives shaped by lived experience; engagement with guest speakers immersed in global health and decolonial research; and reflective discussions about positionality and structural inequities (module evaluation data, 2024–2025). These shifts in classroom climate and participation illustrate how equity-oriented pedagogies can reshape learning environments and support students to develop socially accountable research practices (15).

Faculty involvement both benefits from and amplifies these student experiences. Teaching staff adopt inclusive assessment frameworks, structured feedback, and co-production methodologies, while mentoring clinical fellows and postgraduate tutors extends these practices beyond direct teaching. Faculty participation in national initiatives, including curriculum reform in health professions education, demonstrates how equity-focused programmes can influence institutional culture and sector-wide approaches to applied health research (11).

The programme also highlights structural and systemic challenges. While funded for early-career practitioners within 5 years of professional registration, internationally trained staff, often recruited to address NHS workforce shortages, may face barriers due to residency, fee-status, or post-qualification restrictions (10). This eligibility criteria disproportionately affect minoritised and internationally trained staff, highlighting a tension between workforce realities and research training funding mechanisms (8, 9). Addressing these misalignments is essential if research workforce development initiatives are to advance equity, representation, and social accountability (1).

Key lessons from the programme include:

1. Co-produced research enhances ethical rigor, relevance and applicability of projects (12).
2. Diverse reading lists, case studies and epistemologies, challenge traditional hierarchies of knowledge (14).
3. Mentorship, structured feedback, and reflective supervision, support development for both students and faculty (18).
4. Awareness of structural barriers, particularly for internationally trained and minoritised practitioners, requires flexible policies to reconcile funding criteria with workforce realities (9).

Collectively, these insights demonstrate how equity-oriented research training can foster socially accountable outputs, prepare practitioners to influence policy and practice, and address systemic inequities within the health and care workforce (23).

## Policy and practice implications

1. Flexible eligibility criteria: Expand access for internationally trained practitioners beyond conventional residency or fee-status cut-offs (10).
2. Equity-focused curriculum design: Embed decolonising and social justice principles, diverse epistemologies, co-produced research and culturally responsive pedagogy (24).
3. Mentorship and faculty development: Support faculty through training and mentoring to sustain inclusive practices (11).
4. Community engagement and PPI: Ensure structured involvement of patients, service users, and community stakeholders to enhance relevance and trust (12).
5. Evaluation and reflection: Continuously monitor outcomes, student experiences, and workforce impact to guide iterative improvements (1).

By aligning research training with these principles, the NIHR INSIGHT Programme provides a model for socially accountable, equity-oriented education that prepares practitioners to address health inequities, influence policy and foster inclusive research cultures within the NHS and beyond.

## Limitations

This article presents a reflexive, practice-informed perspective rather than a formal programme evaluation. The insights presented draw on programme documentation, module evaluations, informal feedback, reflective assessments and the authors’ professional experiences as educators and programme leads within the NIHR INSIGHT initiative. As such, the observations reported here are primarily qualitative and context-specific and may not be generalisable to all postgraduate research training programmes.

While student feedback and reflective accounts provide valuable insight into learning environments and classroom dynamics, they do not constitute systematic outcome evaluation. Future research could complement these reflections with longitudinal evaluation of learner outcomes, research outputs and career progression to better understand the longer-term impacts of equity-focused research training initiatives.

Despite these limitations, the perspective offered provides important practice-based insights into how curriculum design, pedagogical approaches and funding structures shape participation in research training and influence the development of socially accountable research capacity within applied health and care professions.

## Conclusion

The NIHR INSIGHT Programme demonstrates how equity-oriented, practice-based research training can support the development of a diverse and socially accountable applied health and care research workforce. By embedding co-produced learning, participatory approaches such as patient and public involvement and critical engagement with structural inequities, the programme creates opportunities for practitioners to connect research training with their professional and community contexts.

At the same time, the programme highlights structural tensions within research workforce development systems. Eligibility criteria tied to residency status, fee classification and conventional professional timelines can unintentionally exclude internationally trained practitioners and other minoritised groups who are already integral to the NHS and social care workforce. Addressing these barriers requires closer alignment between workforce policy, funding frameworks and equity objectives within research training initiatives.

The experiences described in this paper suggest that relatively small but intentional changes in curriculum design, assessment practices and learning environments can shape participation, confidence and engagement in research training. Equity-focused pedagogies, inclusive assessment frameworks and co-produced learning opportunities can help create learning environments where diverse forms of knowledge and lived experience are recognized as valuable contributions to applied health and care research.

As health systems increasingly seek research that is relevant to diverse communities and responsive to complex social determinants of health, programmes such as NIHR INSIGHT provide an important model for integrating research training, professional practice and social accountability. Continued evaluation, policy flexibility and institutional commitment will be essential to ensure that such initiatives fulfill their potential to widen participation and strengthen equity within the health and care research workforce.

Ensuring equitable access to applied health and care research training will require sustained policy commitment, structural investment and inclusive eligibility frameworks to diversify the future research workforce and strengthen socially accountable health research.

## Data Availability

The original contributions presented in the study are included in this article/supplementary materials, further inquiries can be directed to the corresponding author.
